# First interspecific genetic linkage map for *Castanea sativa* x *Castanea crenata* revealed QTLs for resistance to *Phytophthora cinnamomi*

**DOI:** 10.1371/journal.pone.0184381

**Published:** 2017-09-07

**Authors:** Carmen Santos, Charles Dana Nelson, Tetyana Zhebentyayeva, Helena Machado, José Gomes-Laranjo, Rita Lourenço Costa

**Affiliations:** 1 Laboratório de Biologia Molecular, Instituto Nacional de Investigação Agrária e Veterinária, I.P., Avenida da República, Oeiras, Portugal; 2 Southern Institute of Forest Genetics, Southern Research Station, USDA Forest Service, Saucier, Mississippi, United States of America; 3 Forest Health Research and Education Center, University of Kentucky, Lexington, Kentucky, United States of America; 4 Department of Genetics and Biochemistry, Clemson University, Clemson, South Carolina, United States of America; 5 Genomics & Computational Biology Laboratory, Clemson University, Clemson, South Carolina, United States of America; 6 Centro de Investigação e de Tecnologias Agro-Ambientais e Biológicas, Universidade de Trás-os-Montes e Alto Douro, Vila Real, Portugal; 7 Centro de Estudos Florestais, Instituto Superior de Agronomia, Universidade de Lisboa - Tapada da Ajuda, Lisboa, Portugal; Chungnam National University, REPUBLIC OF KOREA

## Abstract

The Japanese chestnut (*Castanea crenata*) carries resistance to *Phytophthora cinnamomi*, the destructive and widespread oomycete causing ink disease. The European chestnut (*Castanea sativa*), carrying little to no disease resistance, is currently threatened by the presence of the oomycete pathogen in forests, orchards and nurseries. Determining the genetic basis of *P*. *cinnamomi* resistance, for further selection of molecular markers and candidate genes, is a prominent issue for implementation of marker assisted selection in the breeding programs for resistance. In this study, the first interspecific genetic linkage map of *C*. *sativa* x *C*. *crenata* allowed the detection of QTLs for *P*. *cinnamomi* resistance. The genetic map was constructed using two independent, control-cross mapping populations. Chestnut populations were genotyped using 452 microsatellite and single nucleotide polymorphism molecular markers derived from the available chestnut transcriptomes. The consensus genetic map spans 498,9 cM and contains 217 markers mapped with an average interval of 2.3 cM. For QTL analyses, the progression rate of *P*. *cinnamomi* lesions in excised shoots inoculated was used as the phenotypic metric. Using non-parametric and composite interval mapping approaches, two QTLs were identified for ink disease resistance, distributed in two linkage groups: E and K. The presence of QTLs located in linkage group E regarding *P*. *cinnamomi* resistance is consistent with a previous preliminary study developed in American x Chinese chestnut populations, suggesting the presence of common *P*. *cinnamomi* defense mechanisms across species. Results presented here extend the genomic resources of *Castanea* genus providing potential tools to assist the ongoing and future chestnut breeding programs.

## Introduction

Chestnuts (Castanea spp., *Fagaceae* family) are found around the world in the temperate zone, where they are highly valued by different cultures for the nutritious nuts, valuable timber and landscaping purposes. European chestnut (*Castanea sativa* Mill.) produces the highest quality and most appreciated nuts, being a major economic income for the European mountainous producing regions and most of that income is being threatened by chestnut depopulation. The emergence of heavily damaging diseases, namely ink disease and chestnut blight, caused by *Phytophthora cinnamomi* and *Cryphonectria parasitica*, respectively are responsible for the decline of the European production from 430,000 Tons to 140,000 Tons in the past 50 years.

*P*. *cinnamomi* is one among the most destructive pathogens associated with the decline of forestry, ornamental and fruit species [1,2]. When introduced to an environment, this pathogen has had enormous impacts on natural systems, and has been shown to reduce the native biodiversity in Europe, the USA, Australia, New Zealand and Africa. In Portugal, ink disease has become widespread, since *P*. *cinnamomi* was first recorded in 1838. In North America, prior to the decimation of the American chestnut (*C*. *dentata* Borkhausen) by chestnut blight, ink disease was partially responsible for its decline in the southeastern part of its range [3]. Although *C*. *sativa* and *C*. *dentata* are susceptible to ink disease and chestnut blight, the Japanese and Chinese chestnuts (*C*. *crenata* Sieb. & Zucc. and *C*. *mollissima* Blume, respectively) show resistance to both diseases [4]. Taking notice of this fact, The American Chestnut Foundation (TACF) have pursued a backcross breeding program, using *C*. *mollissima* as donor parents of resistance, to introgress resistance to *C*. *parasitica* into American chestnut. Nevertheless, ink disease is currently recognized in the USA as a serious threat to the American chestnut restoration program [5]. Consequently, breeding for resistance *to P*. *cinnamomi* was recently initiated in USA [6].

In Portugal, a chestnut breeding program was initiated in 2006 aiming to introgress the resistance from *C*. *crenata* into *C*. *sativa*, by crossing both species. Hybrid progenies, segregating for *P*. *cinnamomi* resistance, have been obtained and extensively studied in order to understand the chestnut resistance mechanisms to ink disease. Accurate phenotyping methodologies were developed to score diverse metrics of resistance of each progeny aiming to identify marker:trait associations [7]. Moreover, a group of hybrid genotypes selected as the most resistant to *P*. *cinnamomi*, are being propagated as improved genetic materials for new rootstocks release to the market in the near future.

Despite the economic and ecological importance of woody plants, mapping of QTLs for important phenotypic traits have been rather limited on these species when compared with major crops or model plants. For chestnut, extensive genomic resources have been developed mainly for *C*. *mollissima* and *C*. *dentata* [8–10]. Nevertheless, the availability of genetic maps and QTLs’ identification is limited for other *Castanea* species. Kubisiak *et al*. (1997) [9] published the first genetic map based on a F2 population of an interspecific cross of *C*. *dentata* and *C*. *mollissima*, where three QTLs for blight resistance were proposed. In 2005, Sisco *et al*. [10], presented an updated version of the genetic map adding 304 markers. Recently, a new genetic map of *C*. *mollissima* was created using 1393 new markers constituting the reference map in which the physical map was anchored [8,11]. So far, only one genetic map was released for *C*. *sativa*, based on a F1 full-sib population of 96 trees, which was used for mapping QTLs for adaptive traits [12,13]. In the present study, the first *C*. *sativa* x *C*. *crenata* genetic map was constructed using markers developed from *C*. *mollissima*, *C*. *dentata*, *C*. *sativa* and *C*. *crenata* transcriptomes. Additionally, QTLs for *P*. *cinnamomi* resistance were detected and mapped for the first time in *C*. *sativa x C*. *crenata*, which is a major breakthrough on a tree which the genome has not yet been sequenced. These new genomic resources should be useful mainly in Europe and in the USA, aiming at improving resistance to ink disease or even for another woody species threatened by *P*. *cinnamomi* such as cork oak, *Quercus suber*.

## Materials and methods

### *C*. *sativa* x *C*. *crenata* mapping populations

Genetic map construction and QTL mapping were carried out using two full-sib families of *C*. *sativa* (female) x *C*. *crenata* (male), obtained from controlled pollination. Genotypic data were collected for 52 progenies from *C*. *sativa* (cultivar Aveleira) x *C*. *crenata2* cross (SxC) and 81 progenies from *C*. *sativa* (cultivar Bária) x *C*. *crenata1* cross (BxC) (Table 1).

**Table 1:** Number of individuals genotyped and markers used in this study.

Individuals genotyped and markers used	SxCpopulation	BxCpopulation	Total
**Nr of individuals genotyped (total)**	52	81	133
**Nr of individuals genotyped (SSRs)**	52	81	133
**Nr of individuals genotyped (SNPs)**	26	0	26
**Nr of polymorphic markers (total)**	435	92	452
**Nr of polymorphic markers (SSRs)**	146	92	180
**Nr of polymorphic markers (SNPs)**	272	0	272

Number of individuals from *Castanea sativa* (cultivar Aveleira) x *C*. *crenata2* (SxC) and *C*. *sativa* (cultivar Bária) *x C*. *crenata1* (BC) crosses and number of polymorphic markers (SSRs and SNPs) used for genetic map construction.

The crosses were performed at the germplasm bank of Universidade de Trás-os-Montes e Alto Douro, Vila Real, Portugal (47°17010″N, 7°44043″W), located at 423 m above sea level on a slope facing southwest stand.

### Phenotyping resistance to *P*. *cinnamomi*

Resistance to *P*. *cinnamomi* was previously measured in Santos et al., (2015) [7]. Progression rate of *P*. *cinnamomi* lesions in inoculated excised shoots was chosen as phenotypic metric as it provided the most differentiation among progenies: from 0.15 cm/day for the most resistant genotype to 1.13 cm/day for the most susceptible one. To obtain these data, excised shoots (the majority with a length of 15 cm) were collected from 45 SxC progenies, in spring and autumn, and were inoculated with an hypervirulent strain of *P*. *cinnamomi* (IMI 340340). The mean number of excised shoots inoculated per genotype was 7.94 in spring and 7.60 in autumn [7].

The external lesion length was measured at five time points after inoculation (5, 7, 9, 12 and 14 days after inoculation) and the lesion progression rates (cm/day) were calculated for each individual, across both seasons [7].

Phenotype experiments were performed in a controlled chamber with temperatures ranging between 18 and 22°C, photoperiod 16 h light/8 h dark and 65% relative humidity.

### Molecular markers source and genotyping

A total of 2972 transcriptome-derived molecular markers developed from four chestnut species (*C*. *dentata*, *C*. *mollissima*, *C*. *crenata* and *C*. *sativa*) (419 simple sequence repeats-SSRs and 2553 single nucleotide polymorphism-SNPs) were screened in the parental genotypes (*C*. *sativa* and *C*. *crenata*). From those, 452 markers (180 SSRs and 272 SNPs) were selected for genotyping the progenies, since they presented polymorphism (at least between two parents from one cross) and a unique amplification product (in case of SSRs) (Table 1). A total of 155 SSRs developed from *C*. *mollissima* (named by CmSI*number*), plus 25 developed from *C*. *sativa* and *C*. *crenata* (named by CsPT*number* and CcPT*number*, respectively) were used [11,14]. SNP markers were developed from *C*. *mollissima* CCall_Unigene_V2 assembly data and from *C*. *dentata* AC454_Unigene_V3 *contig* data (http://www.hardwoodgenomics.org/); they were called as CCallv2*contig number*_*SNP position* and AC454v3*contig number*_*SNP position*, respectively. In this study, in order to simplify the nomenclature, they were named as CC_*contig_number SNP position* and AC_*contig_number SNP position*, respectively. From those SNPs developed from *C*. *mollissima*, 1536 were used for chestnut reference genetic map and were denominated as CmSNP00001-CmSNP01536 [11]. This nomenclature was kept common for SNPs used in this study and in the *C*. *mollissima* reference genetic map. When available, linkage group (LG) information from the reference map (linkage groups A to L) was placed in front of marker name.

For SSR detection, a M13-specific sequence was added to the 5’ end of each forward primer [15] fluorescent dye (6-FAM, NED, PET or VIC) was added to the 5’ end of each forward primer. Loci were amplified individually in 12.5 μl reaction containing: 20 ng of template DNA, 0.16 μM of 5’-dye-labeled M13 primer, 0.04 μM of 5′-tailed forward primer, 0.16 μM of reverse PIG-tailed primer, 66 μM of dNTPs, 2 mM of MgCl_2_, 2 μl 5x GoTaq Flexi Buffer (Promega) and 1.0 U of GoTaq^®^ Flexi DNA Polymerase (Promega). Amplifications were undertaken in a Biometra^®^ T1 Thermocycler using the following profile: 94°C for 2 min; 94°C for 30 s, 60°C (annealing temperature for primer forward and reverse) for 45 s, 72°C for 45 s during 30 cycles; 94°C for 30 s, 53°C (annealing temperature for 5’-dye-labeled M13 primer) for 45 s, 72°C for 45 s during 8 cycles; 72°C for 10 min; indefinite hold at 4°C. Completed reactions were loaded onto an ABI PRISM 310 Genetic Analyzer (Applied Biosystems, Foster City, CA, USA) and run according to the manufacturer’s protocol. Allele sizes were determined using the ROX500 internal size standard and the global southern algorithm implemented by ABI PRISM GeneMapper software version 4.0 (Applied Biosystems).

GoldenGate BeadArray platform and GenomeStudio software were employed for SNP detection, clustering, genotype calling and confidence scores assignment. However, data for all SNPs were inspected manually and edited if necessary [16].

For various logistical reasons only for 26 individuals from the SxC cross were available for SNP genotyping

Total genomic DNA was extracted from 100 mg of fresh leaves using the DNeasy™ Plant Mini Kit (Qiagen, Germany) according to the manufacturer’s instructions.

### Linkage map construction

Marker segregation analyses and linkage map construction were performed using JoinMap v.3.0 software (van Ooijen 2001). Maps were first constructed separately for both mapping families: SxC and BxC, using CP population model. Linkage Groups (LGs) were determined as a logarithm of the odds (LOD score) with a minimum threshold of 4.0. Linkage maps were calculated using the Kosambi mapping function [17] and applying default mapping parameters: all linkage recombination estimates smaller than 0.4 and a LOD larger than 1.0. Syntenic groups among the three maps were identified and combined through the JoinMap “Combine Groups for Map Integration” function. Map orientation was assigned by comparison with *C*. *mollissima* reference map [11]. Linkage groups were drawn using MapChart 2.0 software [18].

### QTL mapping

QTL analyses were performed using MapQTL 5.0 software (Van Ooijen 2004). Utilizing the consensus genetic map created here, QTLs for *P*. *cinnamomi* resistance were detected using SxC population.

Marker significance level was estimated using the Kruskal-Wallis analysis (K-W, non-parametric test); and the interval mapping method was used to detect the presence of a putative QTL [19]. Multiple QTL model (MQM) computation was also applied, using cofactors significantly associated to the trait (P < 0.02), that were estimated by applying a backward elimination procedure (“Automatic cofactor Selection” MapQTL function). The significant LOD threshold (P < 0.05) was estimated from 1000 permutations of the phenotypic trait, for each LG. QTLs were declared when LOD scores (MQM) exceeded the minimum significance threshold for each LG and the associated markers showed K-W test with significance levels above 0.005.

The R^2^ value, representing the percentage of the phenotypic variance explained by the marker genotype at the QTL, was taken from the peak QTL position as estimated by MapQTL software.

Each significant QTL was characterized by the peak marker and the other associated markers within the QTL region, the percentage of phenotypic variation explained (R^2^), LOD score and K-W test. QTL representations were drawn using MapChart 2.0 software [18].

As the molecular markers were developed from transcriptome sequences, putative resistance genes within the QTL intervals genes were identified through BLASTn query against the NCBI database.

## Results

**Fig 1:**
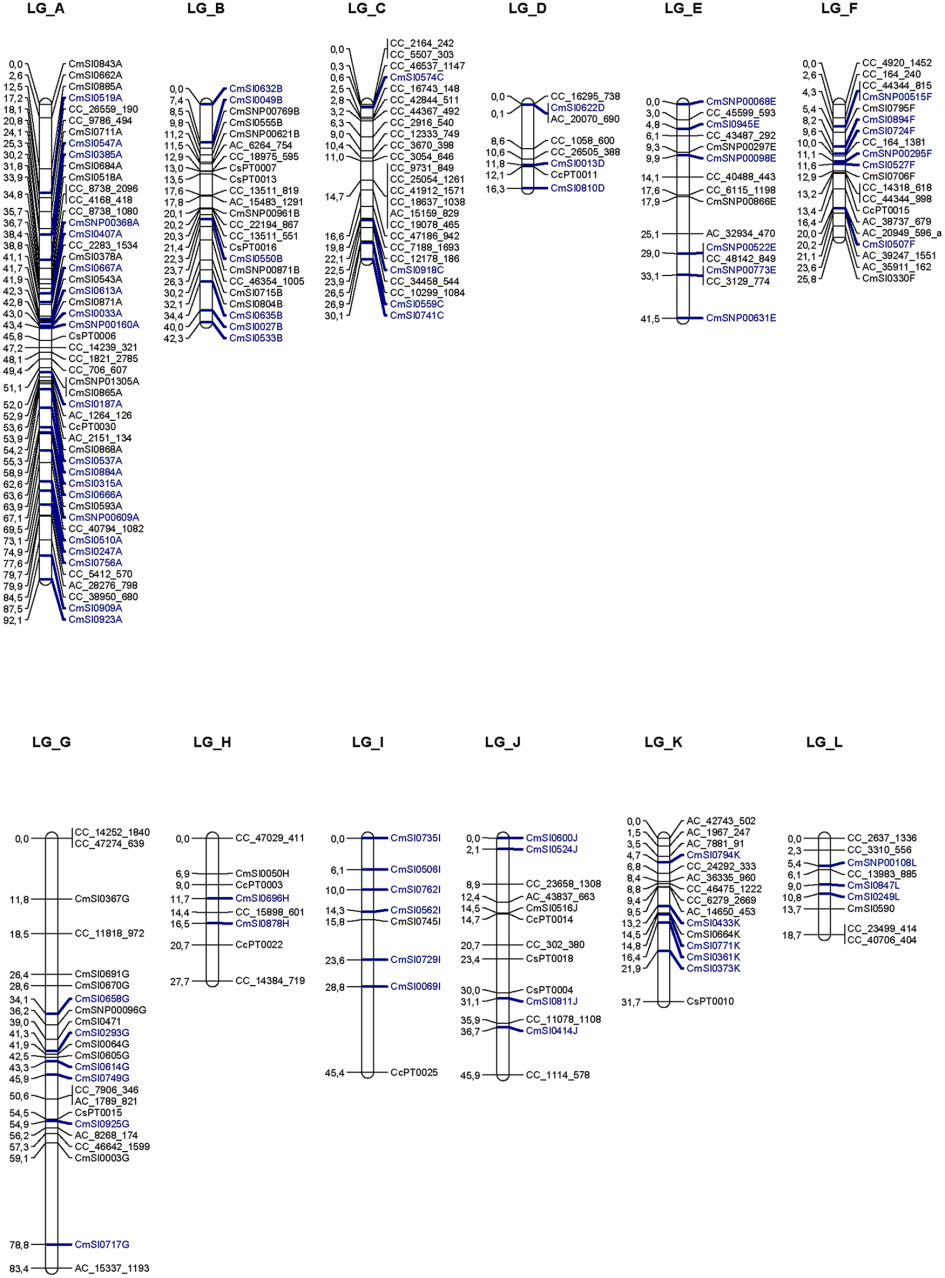
*C*. *sativa* x *C*. *crenata* interspecific consensus genetic map contains 217 markers (SSRs and SNPs) mapped on twelve linkage groups (LGs). Marker positions on LGs were assigned to reference map LGs [11], by using the common markers mapped on both genetic maps (in blue).

The consensus genetic map consists of 217 markers (112 SNPs and 105 SSRs) mapped to 12 LGs, in accordance to chestnut karyotype (n = 12) (Fig 1). According to the genetic reference map [11], all molecular markers developed from *C*. *mollissima* (CmSI*number* and CmSNP*number*) were correctly mapped on each LG; however, not all marker positions are in the same order. Moreover, 25 additional markers (14 SNPs and 11 SSRs) were also grouped to four extra LGs, that were assigned as LG_D1, LG_E1, LG_H1 and LG_J1, according to the genetic reference map (S1 Fig). These groups are included here because they will likely be merged into the consensus map when more progenies/markers are added to this data set. About 34.3% of markers mapped for *C*. *sativa* x *C*. *crenata* were also mapped on the consensus *C*. *mollissima* reference map (Fig 1 and S1 Fig, in blue).

The interspecific genetic map spans a total genetic distance of 498.9 cM (67,3% of genetic distance in the *C*. *mollissima* reference map) with an average interval of 2.3 cM between markers. The genetic length of the linkage groups ranged in size from 16.3 (LG_D) to 92.1 cM (LG_A), with an average of 41.6 cM. From all markers mapped, six (2.7%) of them exhibited poor goodness-of-fit (X^2^ values > 5.0). On the other hand, high segregation distortion was observed for the most of the unmapped markers.

**Fig 2:**
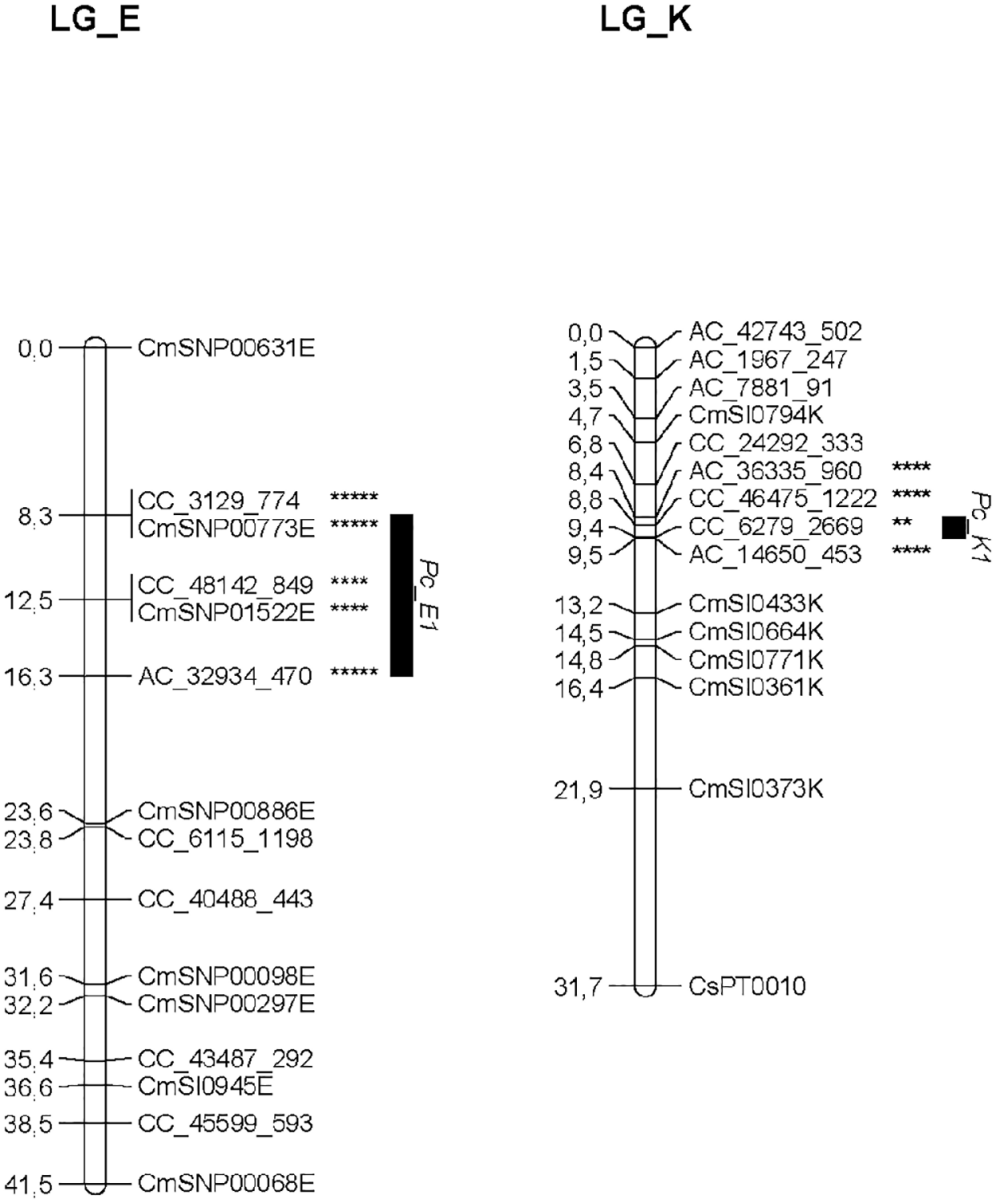
QTLs detected on the linkage groups of the *C*. *sativa* x *C*. *crenata* consensus genetic map by multiple QTL mapping (MQM) for *P*. *cinnamomi* resistance. QTLs are represented by black boxes. Asterisks represents the significance levels obtained by Kruskal-Wallis test: ** = 0.05, **** = 0.005 and ***** = 0.001.

QTL analyses for resistance to *P*. *cinnamomi* was performed for the SxC population where both genotypic and phenotypic data were available. Two QTLs, revealing loci with significant effects (KW test, P<0.005) and LOD scores above the estimated threshold, were identified in two LGs: LG_E and LG_K and therefore the QTL intervals were named *Pc_E*, and *Pc_K*, respectively (Table 2 and Fig 2). The percentage of the phenotypic variance explained by *Pc_E*, and *Pc_K* varied from about 9 to 13%, respectively (Table 2). These QTL intervals were localized to regions about 8 cM and 1 cM in genetic length, respectively. Nine molecular markers (SNPs) in total were associated with the detected QTLs (Table 2 and Fig 2). The strongest QTL marker (based on LOD scores and K-W test) was identified in *Pc_E* showing the highest significance levels in K-W test (α = 0.005 to 0.001).

**Table 2:** Results of MQM mapping for QTLs conferring resistance to *P*. *cinnamomi* in a *C*. *sativa* x *C*. *crenata* population.

				Peak marker					Associated markers				
QTL	LG	Interval (cM)	R^2a^ (%)	Marker	LOD score	Position (cM)^b^	K-W test^c^	Putative function	Marker	LOD score	Position (cM)^b^	K-W test^c^	Putative function
***Pc_E***	E	25.1–33.1	8.8–9.1	CC_3129_774	3.99	33.1	*****	Hormone signalling	CmSNP00773E	3.99	33.1	*****	PAF1 protein
									CC_48142_849	3.71	29.0	****	Resistance protein NDR1/HIN1-Like protein 3
									CmSNP00522E	3.71	29.0	****	Transport of phospholipids
									AC_32934_470	3.80	25.1	*****	Zinc finger, PHD-type
***Pc_K***	K	8.4–9.5	13.3–13.4	AC_36335_960	4.37	8.4	****	Contains a Myb/SANT-like domain	CC_46475_1222	4.36	8.8	****	Uncharacterized
									CC_6279_2669	3.95	9.5	**	Cellulose synthase
									AC_14650_453	3.94	9.5	****	Ribosomal_L6_N

LOD score, position in the LG, Kruskal-Wallis test and putative function of transcript sequences used for marker development are presented for the marker at the LOD peak and for the other associated markers. Asterisks represents the significance levels obtained by Kruskal-Wallis test: ** = 0.05, **** = 0.005 and ***** = 0.001.

Regarding all markers mapped on the QTLs identified, they were developed from transcripts potentially involved in diverse cellular processes. Genes underlying *Pc_E* QTL may be involved in resistance response via NDR1/HIN1-like protein 3 [20], hormone signaling, transport of phospholipids, regulation of gene expression through protein PAF1 homolog and/or Zinc-finger PHD-type (Table 2). On the other hand, the markers associated to *Pc_K* QTL were developed from a EST containing a Myb/SANT domain (involved in regulation of gene expression by alteration of chromatin structure [21]), a cellulose synthase, a ribosomal protein and/or genes with uncharacterized functions to date (Table 2).

## Discussion

Two QTLs associated with resistance to *P*. *cinnamomi* were identified for the first time in a *C*. *sativa* x *C*. *crenata* mapping population, providing an improved understanding about the genetic architecture of pathogen resistance in tree species and especially *Castanea* spp. QTLs were genetically mapped on a new chestnut interspecific linkage map constructed with functional EST-molecular markers, located in transcribed regions (transcriptome-derived). The majority of the markers genotyped are shared with those used for *C*. *mollissima* reference map construction [11], which enabled the confirmation of each LG grouped, increasing the robustness of the presented map. All markers with known position on the reference map were mapped on the same LG presented, providing evidence of strong conservation across *Castanea* species. Furthermore, about 34.3% of markers mapped for *C*. *sativa* x *C*. *crenata* were also mapped on the consensus *C*. *mollissima* reference map. The observation that not all marker positions are in the same order as in the *C*. *mollissima* reference map, may be explained by the presence of more subtle differences between the species, however cytogenetic studies are needed to confirm this supposition.

Combining two full-sib populations from unrelated parents for genetic mapping constitutes a strategy to increase genetic diversity, since native alleles from different species are sampled. The reported genetic map contains 217 molecular markers mapped in 12 LGs, achieving a coverage of 498.9 cM with a marker density of 1 marker / 2.3 cM, on average. In *Quercus robur* and *Q*. *petraea* genetic maps, a similar average of marker density was obtained: 1 marker / 2.9 and 2.7 cM, respectively [22,23]. In chestnut, Casasoli et al. (2001) [12] achieved a *C*. *sativa* genetic map covering 720 cM, and an average of marker density of about 1 marker / 9 cM. A higher density map was expected in this study, since from the 2972 molecular markers available, 452 were polymorphic between two parents in one cross and used for genotyping. The polymorphism rate obtained for *C*. *sativa* x *C*. *crenata* populations (about 15.2%) was higher than polymorphism rate obtained for *C*. *mollissima* population used for constructing the reference map (about 12.6%) [11]. This indicates that the markers developed from *C*. *mollissima* and *C*. *dentata* transcriptomes have a high transferability to the samples of *C*. *sativa* and *C*. *crenata*, mainly for SSRs. Nevertheless, about 44% of the polymorphic markers were not joined to the obtained LGs and therefore were excluded for map construction. Several issues could explain the number of unmapped markers: 1) low number of individuals per population (74 SxC and 81 BxC); 2) missing values, mainly for SNP data, since only 26 individuals were genotyped; but also for SSR data (34% of the 180 SSR markers were excluded from analysis for presenting more than 25% of missing values); 3) marker distortion and 4) absence of segregating markers in some genomic regions, obviating significant linkages between markers with more distant positions. The last hypothesis, could also explain the observation of four extra LGs relative to the expected number (2n = 24, n = 12) (S1 Fig). Concerning the distorted segregation, it appears to be common in interspecific crosses between woody plants. In this study, unmapped markers showed high distortion, probably because of zygotic compatibility issues, which could explain their exclusion from the consensus map. On the other hand, only 2.7% of the mapped markers showed distortion, which is a lower rate when compared with other studies of inter- or intra-specific crosses in *Castanea* and another woody species [9,24–26]. Accordingly, in the *C*. *sativa* genetic map, 10% of the markers showed distortion, however the molecular markers used in that study were RAPD, ISSR and isoenzymes. Indeed, the consensus genetic map constructed for *Quercus* spp. with EST-SSRs showed only 0.9 to 6.8% of distorted markers, depending on the populations [23].

*P*. *cinnamomi* susceptibility was previously evaluated for each SxC progeny, using diverse and accurate methodologies [7]. Due to the recalcitrance of the species, which imposes limitations for *in vitro* establishment and stem cuttings propagation, to produce clonal individuals from each progeny, only 16 SxC progenies were able to be phenotyped, using the standard root inoculation test in biological replicates of each genotype. In the present study, lesion progression rate determined in excised shoots inoculated with the pathogen, was used as phenotypic metric for QTL analyses, because: i) we could have several stems from all progenies; ii) lesion progression rates followed a continuous variation, from 0.15 to 1.13 cm/day, depending on the genotype, enabling the analysis of the trait in a quantitative manner and iii) the results from excised shoot inoculation method were strongly and negatively correlated (-0.85, P<0.001) with the days of survival in root inoculation assays, performed for the subset of SxC progeny [7].

Significant QTLs were detected in two LGs of the consensus map: LG_E and LG_K. The *P*. *cinnamomi* resistance QTL mapped on LG_E was the strongest in our *C*. *sativa* x *C*. *crenata* population. Accordingly, *P*. *cinnamomi* resistance QTL on LG_E were previously reported in a pilot study using two chestnut backcross families (*C*. *mollissima* x *C*. *dentata* hybrid) [6], suggesting a potential important role of those genomic regions in *P*. *cinnamomi* resistance. Moreover, new QTL analyses were performed for recent backcross families obtained in 2014 by TACF, revealing QTLs for *P*. *cinnamomi* resistance again on LG_E and also in LG_K (Tatyana Zhebentyayeva, personal communication). Hence, QTL results obtained for American x Chinese chestnut crosses and European x Japanese chestnut crosses show consistency, indicating that those parents might share resistant haplotypes, located on LG_E and LG_K. These findings suggest common resistance mechanisms to different diseases caused by fungal or fungal-like pathogens across *Castanea* genus as have been recently suggested by Staton et al. (2015) [27].

The QTLs reported in this study, should provide an extensive list of candidate genes for *P*. *cinnamomi* resistance, since the QTL intervals extend to as much as 8 cM. In the future, several resistance candidate genes will be identified in the QTL intervals from chestnut genome (in preparation). Nevertheless, some putative genes underlying the QTLs presented were identified, providing some clues about resistance genes involved in *P*. *cinnamomi* response. Presence of putative resistance proteins, cellulose synthase, regulation of gene expression and hormone signaling may be involved in *P*. *cinnamomi* resistance, as it has been suggested by previous studies of tree-*Phytophthora* interactions [28–31]. The strongest evidence that the QTLs identified here underlie resistance candidate genes, corresponds to the mapping of markers associated to resistance proteins (NDR1/HIN1-Like protein 3) [20] and to the mapping of AC_32934_470 and AC_36335_960 markers on LG_E and LG_K, respectively. These SNPs were developed from genes containing Zinc finger and MYB/SANT domains associated to regulation of gene expression under stress responses [32–34]. Therefore, they may be candidates for downstream studies and applications. In this context, the expression level of transcripts belonging to MYB/SANT and Zinc Finger families have been quantified for three progenies and parents of the breeding program [31]. This recent study showed that *Myb4* gene (containing a MYB/SANT domain) may be involved in the chestnut resistance mechanism in *C*. *crenata* and in hybrid resistant genotypes, by regulating salicylic acid signaling or lignin synthesis in order to avoid pathogen progression [31]. The function of the putative resistance genes underlying the QTLs identified will be confirmed in further research using transcriptomics, proteomics and functional analysis.

Understanding the basic genetic structure of ink disease resistance will increase the accuracy of genomic selection for disease resistance. Since QTL effects vary across environmental and genetic backgrounds, additional populations will be genotyped and phenotyped to validate the QTLs here proposed. Additionally, in the near future, *C*. *sativa* x *C*. *crenata* populations will also be genotyped using a Genotyping-by-Sequencing (GBS) platform, which will increase the map density and consequently the resolution of QTL mapping. The future availability of the chestnut genome sequence will allow the identification of all genetic elements within each *P*. *cinnamomi* resistance QTL.

In summary, the genetic linkage map and QTLs here presented constitute the first effort developed in an interspecific cross between *C*. *sativa* and *C*. *crenata* to map genomic regions associated with *P*. *cinnamomi* resistance and also extend the chestnut genomic resources available. This constitutes a foundation for marker-assisted selection to be applied in the ongoing and future chestnut breeding programs worldwide.

## Supporting information

S1 Fig Four extra LGs obtained for *C*. *sativa* x *C*. *crenata* interspecific cross. Marker positions on LGs were assigned to reference map LGs [11], by using the common markers mapped on both genetic maps (in blue).
(TIF)
