# Supplementary figures and images for: First interspecific genetic linkage map for Castanea sativa x Castanea crenata revealed QTLs for resistance to Phytophthora cinnamomi
Source: PLoS One. 2017 Sep 7;12(9):e0184381. doi: 10.1371/journal.pone.0184381 (PMC5589223; doi:10.1371/journal.pone.0184381)

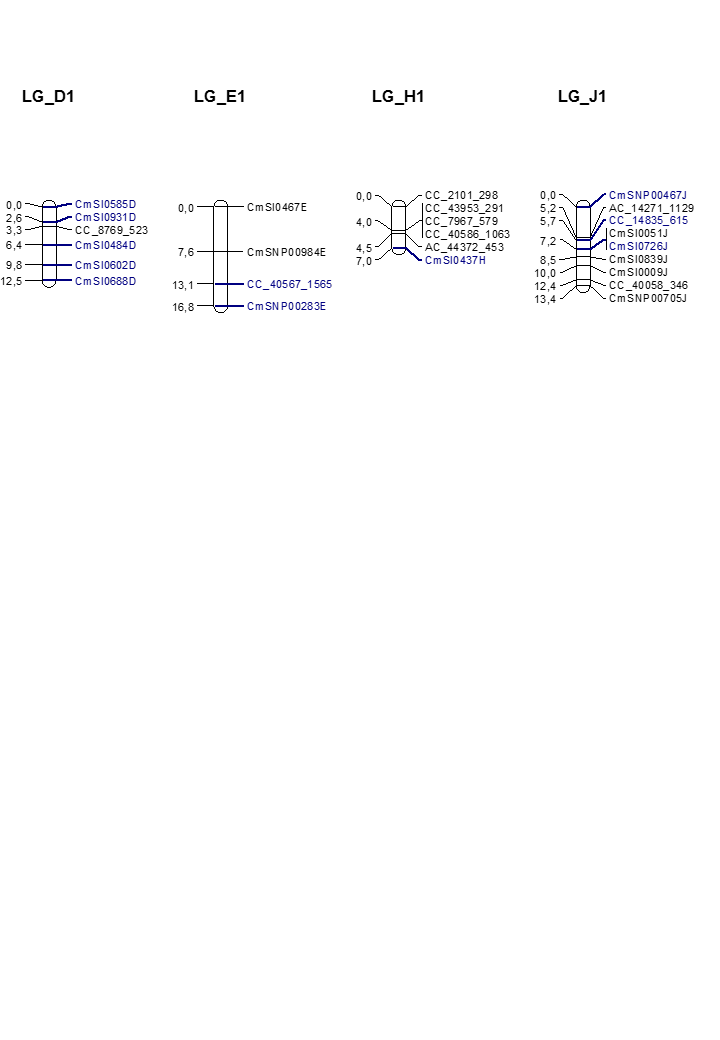

Supplement: S1 Fig — Marker positions on LGs were assigned to reference map LGs [11], by using the common markers mapped on both genetic maps (in blue). (TIF) [file pone.0184381.s001.tif]
